# Advances in Transarterial Radionuclide Therapy for Hepatocellular Carcinoma and Other Liver Tumors: From Yttrium-90 Radioembolization to Emerging Lipiodol-Based Theranostic Approaches

**DOI:** 10.3390/molecules31173030

**Published:** 2026-08-28

**Authors:** Yumiko Kono, Keita Utsunomiya, Shuji Kariya, Noboru Tanigawa

**Affiliations:** Department of Radiology, Kansai Medical University, Hirakata 573-1010, Osaka, Japan

**Keywords:** hepatocellular carcinoma, liver metastases, transarterial radioembolization, yttrium-90, lutetium-177, lipiodol, theranostics

## Abstract

Hepatocellular carcinoma (HCC) remains a leading cause of cancer-related mortality, and internal radionuclide therapy is an important locoregional option for unresectable disease. This review summarizes the current landscape of transarterial radionuclide therapy for HCC and, as a translational perspective, considers how the carrier, radionuclide, and dosimetry concepts established in HCC may extend to secondary liver tumors (liver metastases). It begins with established transarterial radioembolization (TARE/selective internal radiation therapy, SIRT) using yttrium-90 (^90^Y) microspheres and the shift toward personalized dosimetry and combination with systemic and immune-based therapies. It then examines alternative radionuclides and carrier systems—including holmium-166 (^166^Ho) microspheres, iodine-131 (^131^I) lipiodol, rhenium-188 (^188^Re) lipiodol, and lutetium-177 emulsified in lipiodol (^177^Lu-lipiodol)—from the standpoint of their physical properties, radiochemistry, theranostic potential, and preclinical and clinical evidence. Particular attention is given to ^177^Lu-based approaches, including their favorable beta energy, the concurrent imageable gamma emission that enables single-photon emission computed tomography (SPECT)-based dosimetry, and the potential to address post-embolization hypoxia-related radioresistance, while noting that the clinical evidence for ^177^Lu-lipiodol remains preclinical. The application of these platforms to colorectal and neuroendocrine liver metastases is then outlined. Finally, regulatory, radiation safety, and waste management considerations and the principal challenges to clinical translation are discussed.

## 1. Introduction: Principles of Transarterial Radionuclide Therapy

Hepatocellular carcinoma is among the most common causes of cancer death worldwide [1], and many patients are diagnosed at a stage at which curative resection or liver transplantation is not feasible. For these patients, locoregional therapy delivered through the hepatic artery plays a central role. Among such approaches, transarterial radionuclide therapy delivers a high absorbed dose to the tumor while relatively sparing the surrounding non-tumoral liver parenchyma. The same principle underlies transarterial therapy of liver metastases; because the carrier, radionuclide, and dosimetry considerations are largely shared, secondary liver tumors are addressed in this review as a translational extension of the HCC experience. This review summarizes the established role of ^90^Y radioembolization, the alternative and emerging agents under investigation, and the chemical, technical, and regulatory considerations relevant to radiopharmaceutical development, with particular attention to ^177^Lu-lipiodol.

The etiology of HCC shapes the context for these therapies. Chronic hepatitis B and C remain the leading global causes—hepatitis B predominating in East Asia and sub-Saharan Africa, hepatitis C in Japan and Western countries [2,3]—though interferon-free direct-acting antiviral (DAA) therapy has markedly reduced, without eliminating, HCC risk after cure of hepatitis C, so that cirrhotic patients still require surveillance [4,5]. Metabolic dysfunction–associated steatotic liver disease (MASLD) and alcohol-related liver disease are increasingly important and can cause HCC even in non-cirrhotic livers [3]. These shifts affect the patient populations and background liver function relevant to locoregional radionuclide therapy.

This narrative review draws on a PubMed/MEDLINE search of English-language articles (June–August 2026; final search, 11 August 2026) combining the keywords hepatocellular carcinoma, liver metastases, transarterial radioembolization, selective internal radiation therapy, yttrium-90, holmium-166, rhenium-188, iodine-131, lutetium-177, lipiodol, radioembolization-induced liver disease, dosimetry, and theranostics, supplemented by the reference lists of key articles and guidelines and the ClinicalTrials.gov registry. Priority was given to randomized and prospective trials and authoritative guidelines, with preclinical studies included where clinical evidence was limited; as a narrative review, no protocol was registered and no quantitative synthesis was performed.

This therapy exploits a physiological feature of liver tumors: whereas normal hepatic parenchyma is supplied predominantly by the portal vein, HCC nodules derive most of their blood supply from the hepatic artery. A radioactive agent injected selectively into the hepatic artery is therefore preferentially delivered to tumor tissue. Depending on the agent, the therapeutic effect arises from a combination of an embolic component (mechanical occlusion of tumor arterioles) and a radiation component (energy deposited by beta particles within the tumor). The balance between these two mechanisms differs among carrier systems: microspheres act as durable embolic vehicles, whereas lipiodol-based emulsions provide selective tumor retention with comparatively limited macro-embolic effect.

The radiobiological effects of these agents depend on several factors. For the beta-minus (β^−^)-emitting radionuclides considered here, the physically relevant determinants of the absorbed dose distribution include the particle type and its energy spectrum, which together govern the linear energy transfer (LET) and the β^−^ tissue range, the absorbed dose and dose rate delivered, and the physical half-life relative to the biological retention of the carrier. The ultimate biological effect additionally depends on intrinsic cellular radiosensitivity and the tumor microenvironment, which vary among tumors and are incompletely understood. An imageable photon emission does not itself alter the radiobiological effect, but it is of major practical importance because it permits post-administration imaging and image-based dosimetry. These physical factors provide the framework for comparing radionuclides and carriers in the following sections.

## 2. Radionuclides and Carrier Systems

The radionuclides investigated for transarterial therapy of liver tumors span a wide range of beta energies, tissue ranges, and half-lives and differ in whether they emit photons suitable for imaging. This section first summarizes their physical characteristics (Section 2.1) and then the chemical strategies used to incorporate them into clinically usable carriers (Section 2.2), before considering why particular radionuclides are paired with particular carriers.

### 2.1. Physical Characteristics

Table 1 summarizes the representative physical properties of the principal candidate radionuclides. To improve consistency and reproducibility, the tabulated beta energies and photon data are drawn from evaluated decay-data sources (the Decay Data Evaluation Project [DDEP] and the National Nuclear Data Center NuDat 3.0), which share the same evaluated ENSDF basis as the IAEA “Live Chart of the Nuclides” [6,7].

**Figure 1:**
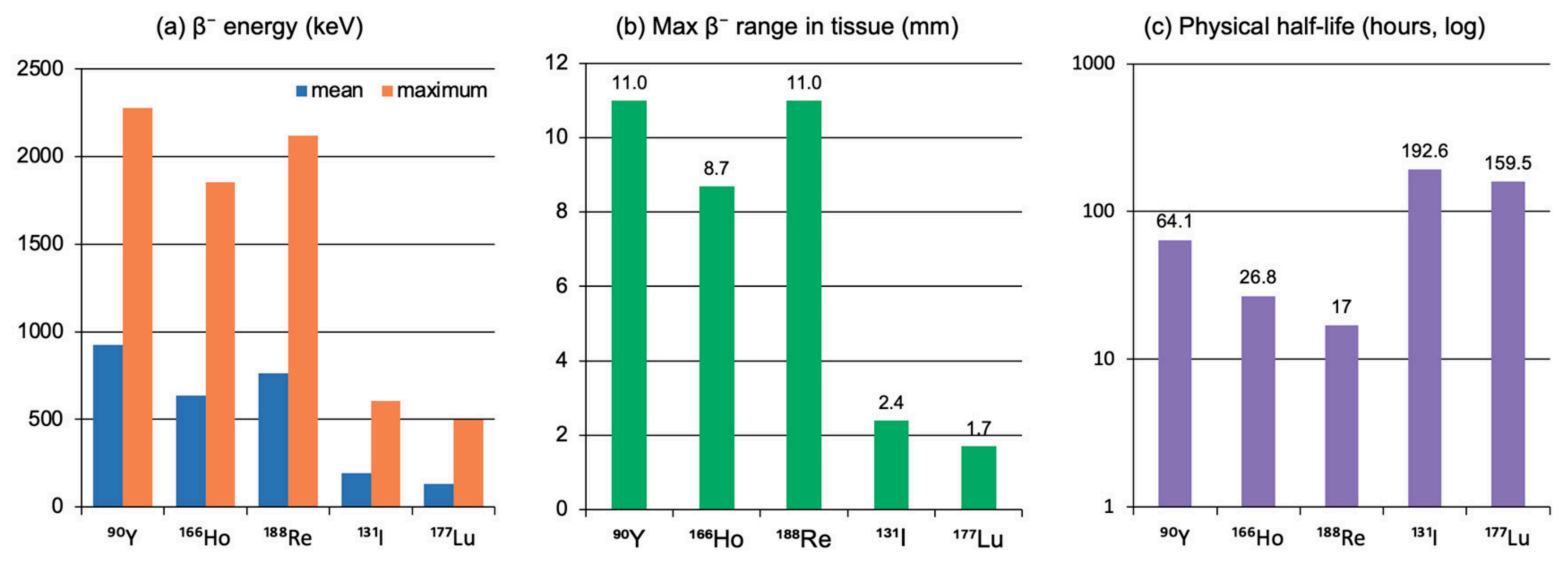
Physical properties of the radionuclides used or investigated for transarterial therapy of liver tumors, standardized from evaluated decay data (DDEP/NuDat 3.0). (**a**) Beta-minus (β^−^) mean and maximum energies; (**b**) maximum β^−^ range in soft tissue (approximate, computed from the endpoint energy); (**c**) physical half-life (logarithmic scale). The same data are presented in tabular form in Table 1.

Table 1 and Figure 1 highlight the practical trade-offs among the agents. ^90^Y provides high-energy beta irradiation but has no primary gamma photon; ^166^Ho and ^188^Re add imageable photons; ^131^I emits an abundant but high-energy, penetrating gamma; and ^177^Lu combines a lower beta energy and short tissue range with low-energy gamma emissions well suited to quantitative SPECT, making it attractive for image-based dosimetry when the carrier remains stably retained in tumor tissue [8,9,10].

^90^Y is a near-pure beta emitter decaying to stable ^90^Zr; its high mean beta energy suits it as a locoregional brachytherapy-like source, but the absence of a primary gamma limits post-treatment imaging to bremsstrahlung SPECT or ^90^Y positron emission tomography (PET) via the small internal pair-production branch (Section 3). ^166^Ho adds an 80.6 keV gamma and—through the large mass of non-radioactive holmium in the microspheres—MRI visibility, so its distribution can be assessed by both scintigraphy and magnetic resonance imaging (MRI; Section 2.2). ^188^Re, eluted from a ^188^W/^188^Re generator, offers high beta energy and a 155 keV imageable gamma and has been used extensively as a low-cost lipiodol label. ^131^I-lipiodol was historically the first widely used transarterial radionuclide agent for HCC [11], but its low beta energy and short range, long 8-day half-life, and abundant, penetrating 364 keV gamma (requiring inpatient management) imposed substantial constraints. ^177^Lu is an attractive theranostic nuclide: a medium-energy beta emitter with a short tissue range and a ~6.6-day half-life, with 113 and 208 keV gammas enabling quantitative SPECT and image-based dosimetry from the therapeutic administration itself.

### 2.2. Chemical Characteristics and Carrier Incorporation

**Figure 2:**
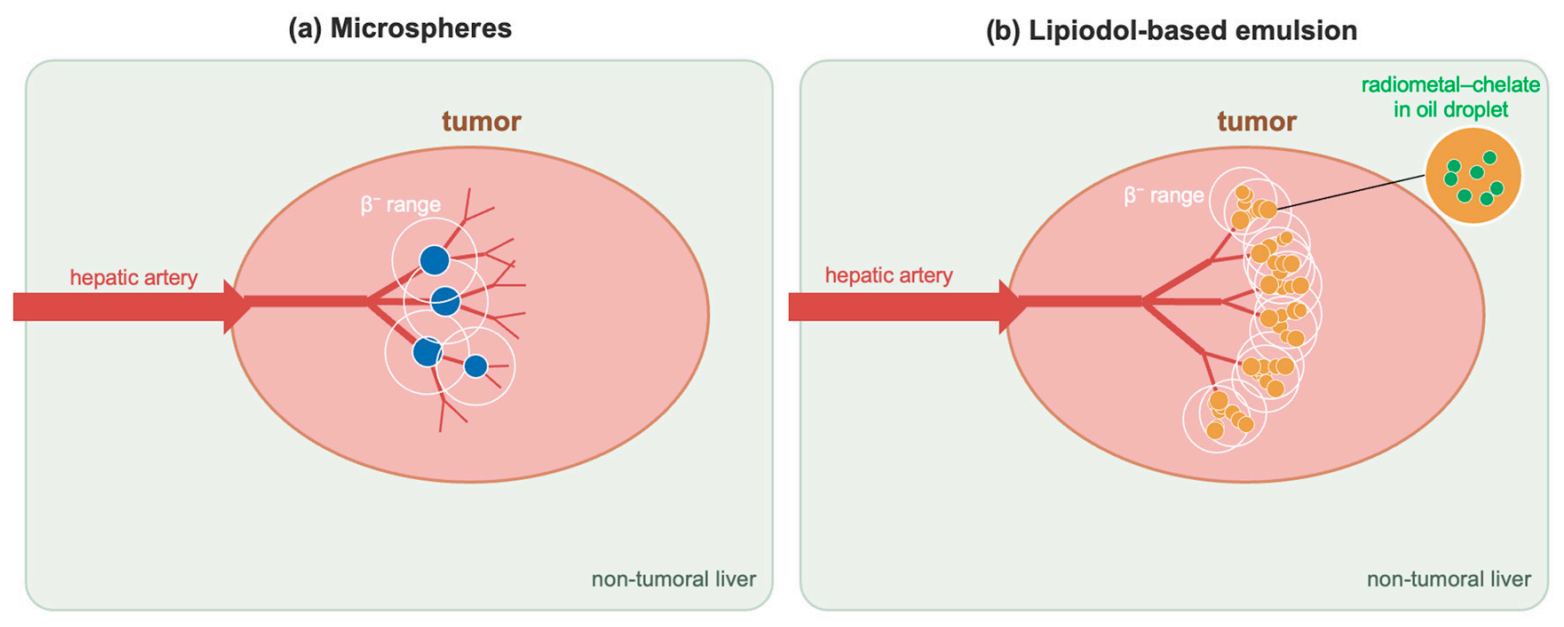
Carrier systems for transarterial radionuclide therapy of liver tumors (schematic, not to scale). The agent is injected selectively into the hepatic artery, exploiting the tumor’s arterial supply versus the portal-venous supply of non-tumoral liver; the red network is the intratumoral vasculature, from larger feeding vessels to distal capillaries. Dashed grey halos denote the β^−^ range in both panels. (**a**) Microspheres (blue) are solid particles embolizing the larger, proximal vessels, not the capillaries. Glass spheres (e.g., TheraSphere) contain ^90^Y neutron-activated in situ from ^89^Y (few, high-activity spheres; lower embolic load), whereas resin spheres (e.g., SIR-Spheres) carry ion-exchange-bound ^90^Y on many low-activity spheres (higher embolic load); ^166^Ho–PLLA spheres are analogous and MRI-visible via the holmium mass. (**b**) The liquid lipiodol emulsion (orange) reaches and embolizes the finest distal vessels, giving a finer, tumor-seeking distribution with limited macro-embolic effect; the inset shows an iodized-oil droplet carrying the radiometal–chelate (green).

Two broad carrier strategies predominate: radioactive microspheres and radionuclides emulsified in lipiodol. Beyond serving as a delivery vehicle, the carrier governs the microdistribution and retention of the agent within the tumor, and the chemistry by which each radionuclide is incorporated differs substantially (Figure 2).

Microsphere carriers are used for ^90^Y (glass or resin spheres) and ^166^Ho (poly(L-lactic acid), PLLA, spheres), providing a controlled particle size, predictable embolic behavior, and a durable intratumoral deposit; the mode of radionuclide incorporation, however, differs by product. In ^90^Y glass microspheres (TheraSphere), ^89^Y is incorporated into the glass and the finished spheres are neutron-activated via ^89^Y(n,γ)^90^Y so that ^90^Y is created in situ as an integral, non-leachable constituent; the high specific activity (thousands of Bq per sphere) means a therapeutic dose uses relatively few particles and a limited embolic load [12]. In ^90^Y resin microspheres (SIR-Spheres), ^90^Y is instead bound to a pre-formed cation-exchange bead, residing on the surface; each sphere carries much lower activity (tens of Bq), so a dose comprises many more particles (tens of millions) with a greater embolic effect and a small potential for radiolabel dissociation [13]. This product-specific difference—rather than a single generic ^90^Y “source”—distinguishes the two products [14]. In ^166^Ho PLLA spheres, non-radioactive ^165^Ho is incorporated by a carrier-added method and partly neutron-activated to ^166^Ho.

A distinctive feature of the ^166^Ho microsphere is that the same particles serve for both therapy and imaging: ^166^Ho emits a SPECT-detectable 80.6 keV gamma, and the spheres are MRI-visible. The MRI signal derives from the paramagnetism of elemental holmium present at high mass concentration—overwhelmingly the non-radioactive ^165^Ho carrier, not the minute quantity of ^166^Ho—so MRI visualizes the microsphere (holmium) distribution rather than the radioactivity itself. Because the spheres can be given at a low (scout) and at therapeutic activity, they support pre-treatment distribution and lung-shunt assessment, detection of extrahepatic deposition, and treatment planning [10,15], as standardized in the EANM procedure guidelines [16].

Lipiodol-based agents require a different strategy. Lipiodol (ethiodized poppy-seed oil) is an iodinated ester selectively retained in HCC after intra-arterial injection—a well-established property underlying its use as a TACE vehicle and tumor-seeking carrier, though retention varies and is greater in well-differentiated, hypervascular tumors than in poorly differentiated tumors or metastases. To exploit it, a radiometal must be made lipophilic and stable enough to partition into, and remain within, the oil phase. ^188^Re-lipiodol has used lipophilic rhenium complexes such as HDD (4-hexadecyl-4,7-diaza-1,10-decanedithiol), SSS (Super-Six-Sulfur), and N-DEDC (nitrido diethyldithiocarbamate) derivatives, with labeling yield and in vivo stability being key determinants of practicality [17,18,19,20]. For ^177^Lu-lipiodol, oxine (8-hydroxyquinoline) carries ^177^Lu into lipiodol; an early formulation showed radiolabel leakage and skeletal deposition [21], whereas a more recent ^177^Lu-oxine/lipiodol emulsion achieved high radiochemical purity (≥99%), durable intratumoral retention on SPECT/CT, and tumor growth suppression in a xenograft model—chiefly through improved purity and in ethiodized poppy-seed oil vivo stability [22].

Thus, radionuclide physics and carrier chemistry must be assessed together, and radionuclide–carrier pairings are not arbitrary. ^90^Y and ^166^Ho are matrix-bound in spheres to provide stable, high-activity embolic sources (and, for ^166^Ho, holmium-based imaging), whereas ^131^I, ^188^Re, and ^177^Lu are emulsified in lipiodol for a finer, tumor-seeking distribution; a free radiometal such as ^90^Y lacks a lipophilic form suited to stable oil-phase retention. Even a favorable radionuclide offers limited clinical value unless the carrier achieves stable arterial delivery, sufficient intratumoral retention, and acceptable off-target redistribution.

Table 2 summarizes the clinical characteristics, imaging options, principal advantages, and translational challenges of the main agents.

## 3. Production, Handling, and Dosimetry

The practicality of each agent depends not only on the radionuclide’s physical properties but also on how it is produced, supplied, handled, and quantified in clinical practice. This section considers production and supply first, then post-administration imaging and dosimetry.

### 3.1. Production and Supply

The candidate radionuclides are produced by distinct routes. ^90^Y is supplied as finished glass or resin microspheres (glass neutron-activated after manufacture, resin surface-labeled with separately produced ^90^Y; Section 2.2). ^166^Ho is reactor-produced by neutron activation of ^165^Ho, incorporated into spheres before activation. ^188^Re is eluted on demand from a long-lived ^188^W/^188^Re generator, making it attractive in decentralized settings. ^177^Lu is reactor-produced directly from ^176^Lu or indirectly via ^176^Yb (ytterbium-176); the route affects specific activity, radionuclidic purity, and the potential long-lived impurity ^177m^Lu [8,9,23]. Specific activity matters for labeling: high-specific-activity or no-carrier-added ^177^Lu helps when chelator or carrier capacity is limited, whereas carrier-added ^177^Lu lowers effective molar activity and may affect labeling for some formulations. For lipiodol-based agents these factors intersect with oil-phase partitioning, emulsion homogeneity, and in vivo dechelation or off-target redistribution.

The physical half-life also governs logistics: the longer-lived ^177^Lu and ^131^I tolerate shipping and flexible scheduling, whereas the short-lived ^188^Re and ^166^Ho require rapid on-site preparation and a tight treatment window. For ^166^Ho, therefore, the constraint is distribution and same-day scheduling imposed by the 26.8 h half-life—the sense in which “supply” is used in Table 2—rather than a shortage of the isotope itself.

### 3.2. Imaging and Dosimetry

Dosimetry is central to efficacy and safety, ranging from mean absorbed dose models—single-compartment (whole-liver) and multicompartment (partition) models distinguishing tumor, non-tumoral liver, and lung—to voxel-level dosimetry from quantitative SPECT or PET. For gamma-emitting agents (^166^Ho, ^188^Re, ^177^Lu), delivery can be quantified and verified from the therapeutic dose itself; ^90^Y, lacking a primary gamma, is imaged by bremsstrahlung SPECT or by PET via its very small internal pair-production branch (≈32 × 10^−6^). ^90^Y PET/CT offers better resolution and quantitative accuracy than bremsstrahlung SPECT and is preferred for dose verification; as typical therapeutic activities (≈1–4 GBq) far exceed the minimum for qualitative imaging (~1 MBq/mL with time of flight, ~3 MBq/mL without, in phantoms) [24], post-therapy ^90^Y PET is generally feasible [25]. The field has moved from fixed prescriptions toward image-based personalized dosimetry, developed in the clinical sections below.

Dose rate considerations differ among radionuclides. Short half-life agents such as ^188^Re and ^166^Ho deliver a large fraction of the absorbed dose over a relatively short interval, producing a high initial dose rate, whereas ^177^Lu provides more protracted irradiation at a lower beta energy. The biological consequences depend not only on physical decay but also on intratumoral retention, subcellular or microvascular distribution, tumor repopulation, and repair of sublethal damage; dose rate arguments should therefore be presented cautiously as mechanistic hypotheses unless supported by carrier-specific dosimetry and outcome data.

## 4. Established Therapy: Yttrium-90 Transarterial Radioembolization (TARE/SIRT)

^90^Y microspheres radioembolization is the most extensively validated form of transarterial radionuclide therapy for HCC and has been in clinical use for more than two decades. Its development has proceeded mainly along two lines: (1) individualized dosing that accounts for each patient’s tumor distribution and liver function and (2) increasingly selective delivery (radiation segmentectomy and radiation lobectomy). In clinical guidelines, the 2022 update of the Barcelona Clinic Liver Cancer (BCLC) classification positions TARE as an option for solitary HCC (≤8 cm) not amenable to, or not responding to, surgical resection or percutaneous treatment. Likewise, the 2023 AASLD (American Association for the Study of Liver Diseases) Practice Guidance positions TARE as an alternative transarterial option to transarterial chemoembolization (TACE) for BCLC stage B HCC.

### 4.1. Personalized Dosimetry and Highly Selective Treatment (Radiation Segmentectomy/Lobectomy)

A landmark trial demonstrating the value of personalized dosimetry was the randomized phase 2 DOSISPHERE-01 trial. In locally advanced HCC, a personalized high-dose approach improved the objective response rate compared with standard dosimetry, and the updated analysis also reported a longer median overall survival [26,27]. Single-arm and retrospective studies have likewise shown good local control with highly selective treatment. The LEGACY study reported a high best objective response rate and durable responses in solitary unresectable HCC [28]. In radiation segmentectomy series, an objective response rate of 90% by European Association for the Study of the Liver (EASL) criteria and durable local control were demonstrated, with 72% of patients free of target lesion progression at five years [29]. More recently, on the basis of the DOORwaY90 study [30], ^90^Y resin microspheres were approved in the United States in 2025 for unresectable HCC. To facilitate comparison across these heterogeneous studies, their design, population, dosimetry approach, principal outcome, and level of evidence are summarized in Table 3.

These findings have shifted ^90^Y TARE practice from fixed-activity prescriptions toward patient-specific dose tailoring, in which a sufficient tumor-absorbed dose is ensured to enhance response while the non-tumoral liver dose is kept within safe limits. In appropriately selected patients—predominantly Child–Pugh A—the use of dosimetry and selective catheter techniques makes symptomatic radioembolization-induced liver disease (REILD; a sinusoidal-obstruction-type liver injury presenting weeks to months after treatment with jaundice, ascites, and hyperbilirubinemia without tumor progression or biliary obstruction) relatively uncommon, reported at around 5% or less [32,33,34].

When interpreting the clinical evidence for ^90^Y TARE, the target population and eligibility criteria of each trial must be specified. The data support selective ^90^Y radioembolization as a potentially curative-intent locoregional treatment in carefully selected patients [26,27,28]; notably, the DOSISPHERE-01 and LEGACY results show high local control even in relatively advanced populations such as locally advanced HCC or solitary unresectable HCC.

### 4.2. A Regulatory Milestone: U.S. Food and Drug Administration (FDA) Approval of SIR-Spheres

In July 2025, the US FDA approved SIR-Spheres Y-90 resin microspheres for local tumor control of unresectable HCC in patients with no macrovascular invasion, Child–Pugh A cirrhosis, well-compensated liver function, and good performance status. This approval was based mainly on the interim analysis of the DOORwaY90 study, in which, among 65 evaluable patients, the best objective response rate by independent central review was 98.5%, all patients with evaluable imaging achieved a local response, and a median duration of response exceeding 300 days was reported [31]. However, this was a single-arm study, and the effect on overall survival and any advantage over systemic therapy remain to be established.

### 4.3. Patient-Related Safety and Complications

^90^Y TARE also has a characteristic complication profile that informs patient selection and technique and differs in part among carriers. Non-target deposition in the gastrointestinal tract via aberrant or reflux vessels can cause radiation gastritis, duodenitis, or ulceration, prevented by careful angiographic technique and, where indicated, coil embolization of at-risk vessels. Hepatopulmonary shunting can irradiate the lungs and, with a high shunt fraction, cause radiation pneumonitis; pre-treatment ^99m^Tc-macroaggregated albumin (or scout-dose) assessment of the lung-shunt fraction is therefore standard, with dose reduction or avoidance above accepted thresholds. Hepatic decompensation and REILD reflect excessive non-tumoral-liver dose—rising with whole-liver or bilobar treatment, limited reserve, and cirrhosis—and are mitigated by selective delivery and partition-model dosimetry [32,33,34]. Biliary strictures, radiation cholecystitis, and biloma or abscess formation can also occur.

These complications differ among carriers. Glass microspheres, carrying high activity on few particles, exert a smaller embolic effect and are often preferred for radiation segmentectomy/lobectomy, whereas the larger particle number of resin spheres gives a greater embolic effect and more flow stasis. Shorter-range emitters such as ^177^Lu-lipiodol would, in principle, spare adjacent parenchyma better, whereas high-energy, long-range ^188^Re delivers more cross-fire; for ^131^I-lipiodol, pulmonary shunting causing radiation pneumonitis was a recognized, sometimes fatal complication contributing to its decline (Section 5). As the emerging lipiodol agents have limited or no clinical safety data, these carrier-specific differences remain partly inferential and require prospective confirmation.

### 4.4. Combination Therapy: Immune Checkpoint Inhibitors and Antiangiogenic Agents

The combination of TARE with systemic therapy—particularly immune checkpoint inhibitors (ICIs) or antiangiogenic agents—is promising but remains investigational. Modulation of the immune microenvironment by TARE (tumor-antigen release, T-cell activation, and induction of checkpoint expression) provides a rationale for combination with ICIs. In a large retrospective dataset, patients with advanced-stage HCC who received TARE plus immunotherapy had better overall survival than those receiving immunotherapy alone; a National Cancer Database analysis reported a median OS of 19.8 versus 9.5 months (adjusted HR 0.50) [35]. However, these results are based on non-randomized data and did not directly compare imaging response rates. Single-arm phase II data reported antitumor activity with an objective response rate (ORR) of 30.6–41.5% for nivolumab after SIRT/^90^Y and 30.8% for a ^90^Y plus pembrolizumab pilot study [36,37]; comparative data establishing an added benefit on tumor response are scarce. TARE plus ICI is therefore best regarded as an investigational strategy in which combining intrahepatic local control with systemic immunotherapy may improve OS or disease-control rate rather than as a combination that clearly increases ORR. Whether the combination is superior to immunotherapy or TARE alone requires prospective comparative trials. Ongoing studies include EMERALD-Y90 [38], which evaluates TARE followed by durvalumab induction and then durvalumab plus bevacizumab, and a ^90^Y plus atezolizumab/bevacizumab trial for pretransplant bridging/downstaging [39].

## 5. Alternative and Emerging Approaches: ^166^Ho, ^131^I, ^188^Re, and ^177^Lu-Lipiodol

The agents in this section span a wide range of evidence maturity, from decades of clinical experience (^131^I-lipiodol) and multinational clinical studies (^188^Re-lipiodol) to a growing microsphere evidence base (^166^Ho) and, at present, purely preclinical data (^177^Lu-lipiodol). To allow a fair comparison, we summarize the clinical evidence and the specific limitations of each of the lipiodol-based agents before discussing ^177^Lu-lipiodol, and we take care to distinguish demonstrated clinical outcomes from physics-based expectations.

### 5.1. Holmium-166 Microspheres

^166^Ho PLLA microspheres were developed to overcome a key limitation of ^90^Y—the difficulty of directly assessing the therapeutic distribution. In addition to beta particles, ^166^Ho emits an imageable 80.6 keV gamma and is MRI-visible owing to holmium’s paramagnetism, so its post-treatment biodistribution can be evaluated by SPECT and MRI, and the same agent can be given at scout and therapeutic activity, reducing discrepancies between simulation and treatment. Because an equivalent dose needs fewer spheres than ^90^Y resin, the risks of flow stasis and reflux are also lower [40]. Its shorter half-life delivers the dose faster; this higher dose rate has been proposed to enhance the biological effect, though its significance awaits validation by dosimetry and outcomes.

Clinical evidence for ^166^Ho microspheres has developed through the HEPAR program. The initial HEPAR phase 1 study established feasibility and dose-escalation safety in patients with unresectable, chemorefractory liver metastases, and the subsequent HEPAR-2 study evaluated efficacy as salvage treatment in patients with liver metastases [15,41]. The HEPAR PLuS study further evaluated additional ^166^Ho radioembolization after ^177^Lu-DOTATATE in neuroendocrine tumor liver metastases [42]. Although these studies mainly involved liver metastases and do not directly demonstrate efficacy in HCC, they provide important evidence for the validity of ^166^Ho as a therapeutic label. The principal HCC-specific evidence comes from HEPAR Primary, a prospective multicenter phase 1/2 study, which showed an acceptable safety profile and early efficacy but, being non-comparative, provides only supportive evidence [43].

Despite these technical advantages, adoption of ^166^Ho radioembolization has been limited by a smaller body of clinical evidence relative to ^90^Y, geographically restricted approval status, and the distribution and scheduling constraints imposed by its short half-life. QuiremSpheres received CE marking in Europe in 2015, and in 2024 the UK National Institute for Health and Care Excellence (NICE) recommended it as an option for unresectable advanced HCC with Child–Pugh A liver function when conventional transarterial therapy is unsuitable (Technology Appraisal Guidance TA985). As dose-response-based individualized treatment and prospective comparative trials in HCC progress, the clinical role of ^166^Ho is expected to become clearer [40].

### 5.2. Iodine-131 Lipiodol

^131^I-lipiodol (iodized oil labeled with ^131^I) was the first transarterial radionuclide agent to enter widespread clinical use for HCC and, unlike the emerging agents discussed below, has been evaluated in randomized controlled trials. In the adjuvant setting after curative resection, a randomized trial reported that a single intra-arterial dose significantly reduced recurrence and prolonged disease-free and overall survival compared with control, leading to early termination for efficacy [44]; longer follow-up showed attenuation of the survival benefit over time [45], and a subsequent randomized trial comparing ^131^I-lipiodol with plain lipiodol confirmed a reduction in intrahepatic recurrence but without a significant difference in overall survival [46]. In unresectable disease, ^131^I-lipiodol was of particular value in patients with portal vein thrombosis, in whom TACE is relatively contraindicated [47], and comparative studies versus chemoembolization showed broadly similar survival with better tolerability of ^131^I-lipiodol, its principal advantage lying in advanced disease and the portal-vein-thrombosis subgroup [48].

Despite this clinical track record, ^131^I-lipiodol has been largely superseded. Its limitations are instructive because they motivate the alternative radionuclides. The abundant, penetrating 364 keV gamma emission necessitates inpatient isolation in a shielded room until the activity decays, a burden compounded by the long 8-day half-life; the relatively low beta energy and short range limit cross-fire in bulky tumors; and pulmonary shunting of lipiodol can cause radiation pneumonitis, a serious and occasionally fatal complication. These factors, together with the commercial withdrawal of the marketed product (Lipiocis) in 2010 following lung toxicity observations, have restricted its current availability, although limited use continues in some countries [49]. Its 364 keV photon, while imageable, is high for standard gamma camera collimators and yields poorer quantitative image quality than the lower-energy emissions of ^177^Lu.

### 5.3. Rhenium-188 Lipiodol

^188^Re-lipiodol has been investigated as an economical, generator-based therapy and—unlike ^177^Lu-lipiodol—has substantial clinical evidence. Multinational, IAEA-supported ^188^Re-HDD studies showed feasibility and efficacy in inoperable HCC: a phase 1 study established dosimetry and tolerability, and a phase 2 study in 185 patients across eight countries reported 1- and 2-year survival of about 46% and 23% [17,18]. They also exposed the central problem—the HDD complex’s in vivo instability, with much activity excreted in urine—motivating more stable complexes. The more stable ^188^Re-SSS complex was tested in the Lip-Re-01 phase 1 activity-escalation study (14 post-sorafenib patients): the maximum tolerated activity was not reached, a recommended activity of 3.7 GBq was defined, and median overall survival was 8.5 months [19]. A ^188^Re-N-DEDC formulation, designed for prolonged retention and kit-based labeling, has reached early clinical evaluation, including in HCC with portal vein thrombosis [20]. A feasibility study using non-selective fixed dosing in Child–Pugh B patients showed substantial hepatic toxicity, underscoring the need for selective delivery and individualized dosimetry [50].

The advantages of ^188^Re-lipiodol are its generator-based, economical production suited to decentralized settings, its imageable 155 keV gamma enabling SPECT and dosimetry, and its high beta energy providing strong tumor cross-fire. Its principal limitations are the short 17 h half-life, which produces a high initial dose rate with rapidly declining tumor irradiation and imposes a tight same-day preparation-and-treatment window, and the radiochemical stability of the rhenium label in lipiodol, which remains the key technical determinant of clinical practicality and the focus of successive complex designs (HDD → SSS, N-DEDC).

### 5.4. Lutetium-177 Lipiodol

Lutetium-177 emulsified in lipiodol (^177^Lu-lipiodol) seeks to combine lipiodol’s selective retention with ^177^Lu’s theranostic properties. ^177^Lu has a medium beta energy (~0.498 MeV maximum) and a short tissue range, favoring localized deposition, and its imageable 113 and 208 keV gammas enable quantitative SPECT and image-based dosimetry after treatment. Relative to the alternatives, these properties suggest three advantages, stated as physics-based expectations rather than demonstrated benefits: the low-energy gammas suit quantitative dosimetry better than the septal-penetrating 364 keV photon of ^131^I; the short beta range could spare non-tumoral parenchyma better than the long-range beta of ^188^Re, relevant in cirrhotic livers with limited reserve; and the intermediate 6.6-day half-life lies between ^131^I (8 d, isolation) and ^188^Re (17 h, same-day-only logistics), potentially allowing central production and shipping with outpatient-compatible radioprotection owing to the low gamma abundance.

Post-embolization hypoxia could limit any embolic β^−^-emitting lipiodol therapy, since embolization induces tumor hypoxia and hence radioresistance. Combining lipiodol’s retention and embolic effect with ^177^Lu’s sustained, image-guided irradiation gives a conceptual rationale for mitigating this through protracted low-dose-rate exposure. Importantly, however, this radioresistance is common to all embolic β^−^ lipiodol agents and is not specific to ^177^Lu; whether ^177^Lu’s protracted irradiation confers any advantage here remains to be tested.

This concept is supported by a preclinical xenograft study from our group [22]. ^177^Lu-oxine synthesized from ^177^Lu-chloride was emulsified in lipiodol at high radiochemical purity (≥99%) and administered intra-arterially to HC-4 tumor-bearing rats. Serial SPECT/CT confirmed durable tumor accumulation, and growth of the treated tumors was significantly suppressed compared with non-labeled lipiodol controls. Tumor volume decreased until day 21 but showed partial regrowth by day 28, suggesting that repeated administration may be required for durable control. A complementary in gamma-camera collimators in vitro irradiation experiment confirmed a dose-dependent reduction in HC-4 cell proliferation, supporting their radiosensitivity. Off-target uptake was limited apart from moderate splenic accumulation, and assessment of hematologic toxicity remains a future task.

These findings support ^177^Lu-lipiodol as a rational next-generation candidate. However, it is essential to recognize that the current evidence is entirely preclinical, with no published human efficacy or survival data, whereas ^131^I- and ^188^Re-lipiodol have been evaluated clinically, including in randomized (^131^I) and multinational (^188^Re) studies. The physics-based advantages outlined above should therefore be regarded as hypotheses that motivate clinical investigation rather than as established superiority. Clinical translation will require clarifying radiolabel stability, repeated-dosing design, hematologic toxicity, normal-organ dose, and the relationship between SPECT-based dosimetry and therapeutic effect, and ideally direct comparison with the existing lipiodol-based agents.

PSMA-targeted strategies are also being explored in HCC, although they are not transarterial. PSMA expression can occur in tumor neovasculature and in some non-prostatic malignancies, and recent preclinical/translational work with ^177^Lu-PSMA-617 supports the feasibility of PSMA-targeted imaging and radioligand therapy models for HCC [51]. ^177^Lu-PSMA therapy for HCC remains exploratory and should be discussed separately from transarterial radioembolization, but it may constitute a new area of investigation.

## 6. Extension to Liver Metastases (Translational Perspectives)

This section outlines how the carrier, radionuclide, and dosimetry concepts of transarterial radionuclide therapy developed in HCC extend to secondary liver tumors. Below, colorectal liver metastases (CRLMs), for which randomized evidence has accumulated, and neuroendocrine tumor liver metastases (NET-LMs), which have a strong affinity with theranostics, are each addressed as translational examples.

### 6.1. Similarities and Differences with HCC

The physiological rationale described above—that normal parenchyma is perfused predominantly by the portal vein while tumors derive their blood supply preferentially from the hepatic artery—is broadly common to many liver metastases as well as HCC. The same treatment platforms (microspheres and lipiodol-based carriers, imageable theranostic radionuclides, and image-based dosimetry) may therefore be applied to liver metastases. However, liver metastases differ from HCC in several important aspects that affect treatment strategy and the interpretation of evidence. First, liver metastases are often accompanied by a primary tumor or extrahepatic disease, so combination with systemic chemotherapy or molecular-targeted agents is a standard premise. Second, the background liver is often non-cirrhotic with relatively preserved functional reserve, so the latitude for dose design, the tolerance of repeated administration, and the nature of REILD may differ from HCC. Third, tumor biology differs markedly by primary tumor, and treatment goals and levels of evidence differ substantially between CRLMs and NET-LMs.

### 6.2. Colorectal Liver Metastases: An Overview of Randomized Evidence

CRLM is the area with the most accumulated randomized evidence for ^90^Y radioembolization of liver metastases. The combined SIRFLOX/FOXFIRE/FOXFIRE-Global analysis evaluating the addition of radioembolization to first-line chemotherapy did not show prolongation of overall survival, although SIRFLOX showed a significant prolongation of liver-specific progression-free survival (control, 12.6 months vs. SIRT, 20.5 months). The EPOCH trial, evaluating combination with second-line therapy, significantly improved progression-free survival and hepatic progression-free survival (PFS hazard ratio, 0.69), whereas overall survival was not improved. These results indicate that ^90^Y radioembolization in CRLM may contribute to control of hepatic disease, but an additive effect on overall survival has not been established and patient selection is important [52,53].

### 6.3. Neuroendocrine Tumor Liver Metastases: Liver-Directed Therapy and Theranostics in the Peptide Receptor Radionuclide Therapy (PRRT) Era

NET-LM is the area most closely related to the discussion of theranostics and carrier systems in this review. ^90^Y radioembolization is positioned in multiple clinical studies and reviews as an effective liver-directed treatment for NET liver metastases [54,55], and it is notable for being combinable with PRRT (^177^Lu-DOTATATE), a systemic theranostic therapy targeting somatostatin receptors. The HEPAR PLuS study, a prospective phase 2 trial adding ^166^Ho radioembolization after PRRT, reported a per-protocol objective response rate of 43% in the treated liver, embodying the concept of a dose boost that combines systemic theranostic therapy with a liver-directed theranostic radionuclide [42]. In addition, intra-arterial PRRT (IA-PRRT), in which ^177^Lu-DOTATATE is administered directly into the hepatic artery, is being explored as a strategy to increase tumor dose while limiting normal-organ exposure. However, these remain single-arm, small-series, or investigational, and confirmation by randomized comparison is a future task.

### 6.4. Implications from the Carrier/Radionuclide Perspective

Viewing liver metastases from the carrier/radionuclide perspective reveals several points of contact with the main theme of this review. First, in liver metastases, the background liver is often non-cirrhotic with relatively preserved functional reserve, which may allow greater latitude in dose design and tolerance of repeated administration than in HCC. This could be a favorable clinical context for agents such as ^177^Lu-lipiodol, for which durable control may be difficult to achieve with a single treatment and repeated administration is anticipated. Second, NET liver metastases have a strong affinity with imageable theranostic radionuclides, particularly ^177^Lu and ^166^Ho. ^166^Ho allows distribution to be confirmed by SPECT and MRI and enables pre-treatment individualized dosimetry with a low scout activity, whereas ^177^Lu enables image-based dosimetry through quantitative SPECT after the therapeutic administration. When PRRT is combined with liver-directed therapy, absorbed doses from systemic and liver-directed administration must be assessed in an integrated manner, further increasing the importance of image-based dosimetry and individualized treatment planning. Taken together, the emerging approaches discussed in this review, including ^177^Lu-lipiodol, may merit investigation not only in HCC but also in liver metastases, particularly NET liver metastases. However, no direct clinical evidence exists for ^177^Lu-lipiodol in liver metastases at present, and this direction remains a conceptual perspective.

## 7. Theranostics, Regulatory, and Radiation Safety Considerations

### 7.1. Theranostics and Future Directions

A major advantage of ^177^Lu and ^166^Ho is that the therapeutic agent itself can be imaged, enabling pre-treatment patient selection, post-administration verification of agent distribution, and image-based personalized dosimetry. This theranostic property is important for advancing transarterial radionuclide therapy toward dose-response-guided, individualized treatment. Future directions include quantitative imaging biomarkers, voxel-level dose-response analysis, and the application of computational and machine-learning methods to dosimetry and outcome prediction.

### 7.2. Regulatory, Radiation Safety, and Waste Management Considerations

Because these therapies use unsealed sources, their clinical implementation is governed by radiation protection regulation as much as by oncologic evidence. Relevant considerations include the handling and shielding requirements that follow from each radionuclide’s photon emissions and half-life, patient-release criteria, and the management of radioactive waste generated during preparation and administration. As discussed in Section 4.3 and Section 5, these radiation protection requirements are inseparable from patient safety: for example, the penetrating 364 keV gamma of ^131^I that mandates inpatient isolation is the same emission that constrained its clinical use. Regulatory frameworks for radioactive medical waste differ internationally—for example, between decay-in-storage approaches and clearance-based systems—and these differences materially affect the practical adoption of agents with longer half-lives such as ^177^Lu. The viability of a radiopharmaceutical therefore depends not only on its radiochemistry and radiobiology but also on whether it can be produced, administered, and disposed of within the prevailing regulatory environment.

Radiation safety and waste management requirements can determine whether a promising radiopharmaceutical is practically deployable. Relevant variables include physical half-life, gamma emission, administered activity, residual activity in vials and catheters, patient excreta, contamination risk during emulsion preparation, and whether the product contains long-lived impurities. For ^177^Lu, the potential presence of ^177m^Lu after direct production is particularly relevant because its much longer half-life can complicate decay-in-storage strategies [23,56].

Regulatory frameworks differ by jurisdiction. In the United States, decay-in-storage procedures for certain medical radioactive waste are addressed under Nuclear Regulatory Commission regulations and guidance, whereas European practice is shaped by the Euratom Basic Safety Standards and national implementation. Patient-release criteria, waste contractor acceptance, storage capacity, and documentation requirements also vary among institutions [57,58].

Accordingly, advancing a relatively long-lived, imageable radionuclide such as ^177^Lu toward clinical use requires an integrated assessment of regulatory compliance—encompassing production, administration, patient management, and waste disposal—in addition to its radiochemical and radiobiological properties.

## 8. Conclusions

Transarterial radionuclide therapy of the liver, led by ^90^Y radioembolization for HCC, has evolved from a primarily palliative technique into a personalized, dosimetry-guided treatment, and its carrier, radionuclide, and dosimetry concepts extend naturally to secondary liver tumors. Emerging agents such as ^166^Ho microspheres, ^188^Re-lipiodol, and ^177^Lu-lipiodol use imageable theranostic radionuclides to enable verification of post-treatment distribution and image-based dosimetry. Clinical translation of ^177^Lu-lipiodol and other strategies will require improved label stability, standardization of dosimetry, validation in prospective clinical trials, and the development of appropriate regulatory and radiation safety frameworks. The integration of radiopharmaceutical chemistry, quantitative imaging, and personalized dosimetry is likely to drive the next phase of progress in this field.

## Figures and Tables

**Table 1:** Physical properties of radionuclides used or investigated for transarterial therapy of liver tumors. All beta values refer to beta-minus (β^−^) emission. Mean and maximum β^−^ energies and gamma intensities are taken from evaluated decay data (DDEP/NuDat 3.0); maximum soft-tissue ranges are approximate values computed from the endpoint energy and are given to indicate order of magnitude only.

Radionuclide	Physical Half-Life	β^−^ Mean Energy (keV)	β^−^ Max Energy (keV)	Max Soft Tissue Range (mm) ^a^	Imageable Emission
^90^Y	64.1 h	927	2279	11 mm (mean, 2.5)	None (pure β^−^); imaged by bremsstrahlung SPECT or PET via internal pair production (511 keV, ~3.2 × 10^−5^/decay)
^166^Ho	26.8 h	≈635	1854	8.7 mm (mean, ≈2.1)	80.6 keV γ (6.6%); tissue distribution is also MRI-visible (see Section 2.2)
^188^Re	17.0 h	≈764	2120	11 mm (mean, 2.5)	155 keV γ (15.6%)
^131^I	8.02 d	192 ^b^	606 ^b^	2.4 mm (mean, ≈0.4)	364 keV γ (81.2%)
^177^Lu	6.647 d	≈133	498	≈1.7 mm (mean, ≈0.23)	208 keV γ (10.4%); 113 keV γ (6.2%)

^a^ Maximum β^−^ range in soft tissue/water, computed (continuous-slowing-down approximation); approximate and convention-dependent. ^b^ For ^131^I, the values given are for the dominant β^−^ branch (endpoint, 606 keV, 89% intensity; mean, 192 keV), which is the dosimetrically relevant emission. The absolute β^−^ endpoint of ^131^I is 807 keV, but this belongs to a minor branch (~0.4% intensity) and is not representative of the therapeutic beta spectrum.

**Table 2:** Clinical characteristics of the main agents used or investigated for transarterial radionuclide therapy of liver tumors.

Agent	Carrier	Target	Development Stage	Imaging	Key Advantages	Key Challenges
^90^Y microspheres	glass/resin	HCC, metastases	Established (FDA-approved *)	PET, bremsstrahlung SPECT	Robust evidence base	No primary γ; dosimetry
^166^Ho microspheres	PLLA	HCC, metastases	Mainly Europe (NICE-recommended)	SPECT, MRI	Scout dose; MRI-visible	Short half-life; on-site scheduling and distribution logistics
^131^I-lipiodol	lipiodol	HCC	Clinical (incl. RCTs); largely superseded/withdrawn	SPECT (364 keV)	Clinical evidence incl. RCTs; tumor-seeking	Long half-life; penetrating 364 keV γ requiring inpatient isolation; commercial withdrawal
^188^Re-lipiodol	lipiodol	HCC	Early/phase 1–2 clinical	SPECT (155 keV)	Generator-based, economical; imageable	Label stability; short half-life; on-site prep
^177^Lu-lipiodol	lipiodol	HCC	Preclinical	SPECT (113/208 keV)	Image-based dosimetry; short β^−^ range	No clinical data yet

Development/approval status as of 2026. * Indications differ by product and country. FDA: Food and Drug Administration; NICE: National Institute for Health and Care Excellence; PLLA: poly(L-lactic acid).

**Table 3:** Representative clinical studies of ^90^Y TARE for HCC. Reported outcome figures are drawn from the cited primary publications. DOSISPHERE-01 response and survival figures are for the modified intention-to-treat (treated) population; the DOORwaY90 figures are from the interim analysis (conference/regulatory data), the trial’s full primary results manuscript being awaited.

Study [Ref.]	Design	Population	*n*	Dosimetry/Approach	Primary Endpoint	Principal Outcome	Level of Evidence
DOSISPHERE-01 [26,27]	Randomized, open-label phase 2	Locally advanced HCC (≥1 lesion ≥7 cm), Child–Pugh A	60 randomized (56 treated)	Personalized vs. standard dosimetry	Objective response rate	ORR 71% vs. 36% (mITT); updated median OS 24.8 vs. 10.7 mo	Randomized phase 2
LEGACY [28]	Retrospective, multicenter, single-arm	Solitary unresectable HCC ≤ 8 cm	162	Radiation segmentectomy (high tumor dose)	Best objective response, duration of response	BOR 88.3%; durable responses; 3 yr OS 86.6%	Retrospective cohort
Radiation segmentectomy [29]	Retrospective, single-center	Early/solitary HCC	≈70	Segmental high-dose delivery	Response/local control	ORR 90% (EASL); 72% free of target lesion progression at 5 yr	Retrospective cohort
DOORwaY90 [30,31]	Prospective, multicenter, single-arm (interim)	Unresectable HCC, Child-Pugh A	65 (evaluable)	Personalized dosimetry, resin microspheres	Best objective response rate	ORR 98.5% (independent review); median DoR > 300 d	Single-arm phase 2 (interim)

## Data Availability

No new data were created or analyzed in this study. Data sharing is not applicable to this article.
